# Supplementary figures and images for: LncRNA H19 Promotes Lung Adenocarcinoma Progression via Binding to Mutant p53 R175H
Source: Cancers (Basel). 2022 Sep 16;14(18):4486. doi: 10.3390/cancers14184486 (PMC9496924; doi:10.3390/cancers14184486)

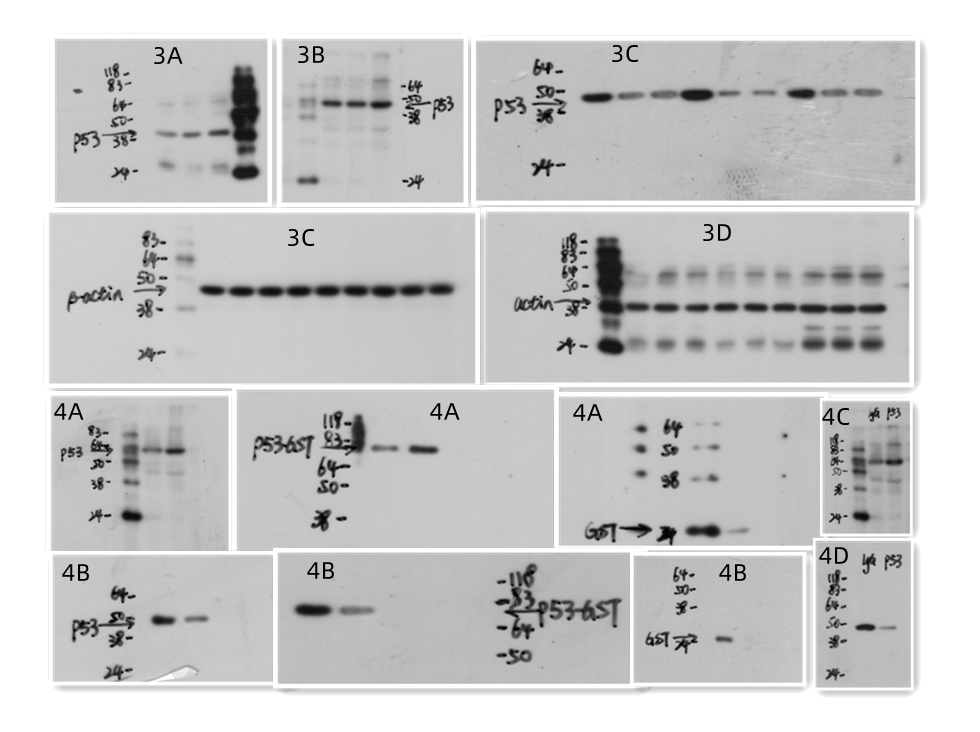

Supplement: Supplementary file 1 [file cancers-14-04486-s001.zip › cancers-1799501-supplementary.png]
